# Comprehensive characterization and molecular insights into the salt tolerance of a Cu, Zn-superoxide dismutase from an Indian Mangrove, *Avicennia marina*

**DOI:** 10.1038/s41598-022-05726-6

**Published:** 2022-02-02

**Authors:** Rajat Kanti Sarkar, Moumita Bhowmik, Moumita Biswas Sarkar, Gaurab Sircar, Kashinath Bhattacharya

**Affiliations:** 1 Department of Botany, Siksha Bhavana, Visva-Bharati (A Central University), Santiniketan, West Bengal 731235 India; 2 Division of Plant Biology, Bose Institute, Kolkata, West Bengal 700009 India

**Keywords:** Enzyme mechanisms, Enzymes, Proteins, Protein folding, Plant biotechnology, Plant biotechnology, Plant molecular biology, Plant stress responses

## Abstract

Superoxide dismutases are important group of antioxidant metallozyme and play important role in ROS homeostasis in salinity stress. The present study reports the biochemical properties of a salt-tolerant Cu, Zn-superoxide from *Avicennia marina* (*Am*_SOD). *Am*_SOD was purified from the leaf and identified by mass-spectrometry. Recombinant *Am*_SOD cDNA was bacterially expressed as a homodimeric protein. Enzyme kinetics revealed a high substrate affinity and specific activity of *Am*_SOD as compared to many earlier reported SODs. An electronic transition in 360–400 nm spectra of *Am*_SOD is indicative of Cu^2+^-binding. *Am*_SOD activity was potentially inhibited by diethyldithiocarbamate and H_2_O_2_, a characteristic of Cu, Zn-SOD. *Am*_SOD exhibited conformational and functional stability at high NaCl concentration as well in alkaline pH. Introgression of *Am*_SOD in *E. coli* conferred tolerance to oxidative stress under highly saline condition. *Am*_SOD was moderately thermostable and retained functional activity at ~ 60 °C. In-silico analyses revealed 5 solvent-accessible N-terminal residues of *Am*_SOD that were less hydrophobic than those at similar positions of non-halophilic SODs. Substituting these 5 residues with non-halophilic counterparts resulted in > 50% reduction in salt-tolerance of *Am*_SOD. This indicates a cumulative role of these residues in maintaining low surface hydrophobicity of *Am*_SOD and consequently high salt tolerance. The molecular information on antioxidant activity and salt-tolerance of *Am*_SOD may have potential application in biotechnology research. To our knowledge, this is the first report on salt-tolerant SOD from mangrove.

## Introduction

Oxidative stress in aerobic organisms refers to the production of oxygen byproducts and is triggered by various environmental factors. The most immediate response to these environmental stresses is the enhanced level of free radicals that include singlet oxygen (^1^O_2_), hydrogen peroxide (H_2_O_2_), superoxide radical (O2^−·^), hydroxyl radical (OH^·^), hydroperoxyl/perhydroxyl radical (HO_2_^·^), alkoxy radical (RO^·^), peroxy radical (ROO^·^) and excited carbonyl (RO^*^). These reactive oxygen species (ROS) can lead to potential damage at cellular as well as genetic level leading to detrimental effects such as cell death and DNA mutation^1^. In living cells, the antioxidant system is crucial for combating cellular oxidative stress^2^. Superoxide dismutase (SOD; EC 1.15.1.1) is an essential component of this antioxidant system to provide first-line enzymatic defense by catalyzing the dismutation of superoxide radicals into O_2_ and H_2_O_2_ at a diffusion-limited catalytic rate^3^. Depending on the enzyme-bound metal co-factors, four different forms of SOD exist such as Cu, Zn-SOD and Fe-SOD (chloroplasts, cytosol, mitochondria, peroxyzome), Mn-SOD (mitochondria, peroxyzome), and Ni-SOD (prokaryotic cytosol). Such specific subcellular location of each isoform is thought to be important for compartmentalized redox signaling.

Among all SOD enzymes, Cu, Zn-SOD is the most abundant type and is mostly localized in the cytosol, chloroplast, peroxisome, and sometimes in extracellular spaces^4^. Cu, Zn-SOD mostly exists in homo-dimeric form with non-covalently attached Cu and Zn ions in each subunit^5^. While the zinc ion was found to be responsible for stabilizing the SOD dimer, the copper ion, via an alternate oxidation-reduction mechanism, helps this enzyme to catalyze a two-step superoxide dismutation reaction^6^. Certain Cu, Zn-SODs were also found to exhibit unaltered catalytic activity even in the presence of ionic detergents, chaotropic agents, extreme pH, and high temperature^7–9^. Because of its cellular abundance, diverse organellar distribution, high kinetic stability, and oxidative stress tolerance property Cu, Zn-SOD has become a good candidate for biochemical studies and subsequent biotechnological applications. Some highly stable SOD enzymes have been reported from a wide range of extremophilic organisms like archaea, extremophilic bacteria, xerophytes, and halophytes^10–14^. Halophytic adaptations are commonly found in mangrove plants which are continuously challenged with salinity stress. The ROS homeostasis in these mangroves is performed by robust antioxidant system including components such as SOD. Certain stress-combating enzymes from halophilic organisms have also been found functionally stable in highly saline microenvironments^15–17^. Three major species under the genus *Avicennia* have been reported to be predominant constituents of Indian mangrove flora^18^. Some studies have been performed to understand the mechanism of combating oxidative stress under highly saline conditions^19–22^. A transgenic experiment was done in which introgression of Cu, Zn-SOD gene from *Avicennia marina* into rice resulted in enhanced salt tolerance^23^. A recent study was performed on a Cu, Zn-SOD isolated from *Avicennia marina* growing in the mangrove forest of the Middle East coast^24^. The study was a preliminary report that revealed a noticeably high halo-tolerance of this enzyme as established by some biochemical assays. However, a detailed understanding of the molecular basis of the high antioxidant activity of this enzyme in presence of high salt concentration is not yet available.

In this communication, we report a full-length Cu, Zn-SOD enzyme (*Am*_SOD) isolated from the *Avicennia marina* of Indian Sundarban. A recombinant expression followed by a comprehensive biochemical, and biophysical characterization of *Am*_SOD was done. We also present here a deeper insight into salt-tolerant features of this enzyme at residue level through a rational mutagenesis approach.

## Results

### *Avicennia marina* showed the highest superoxide dismutase activity

**Figure 1:**
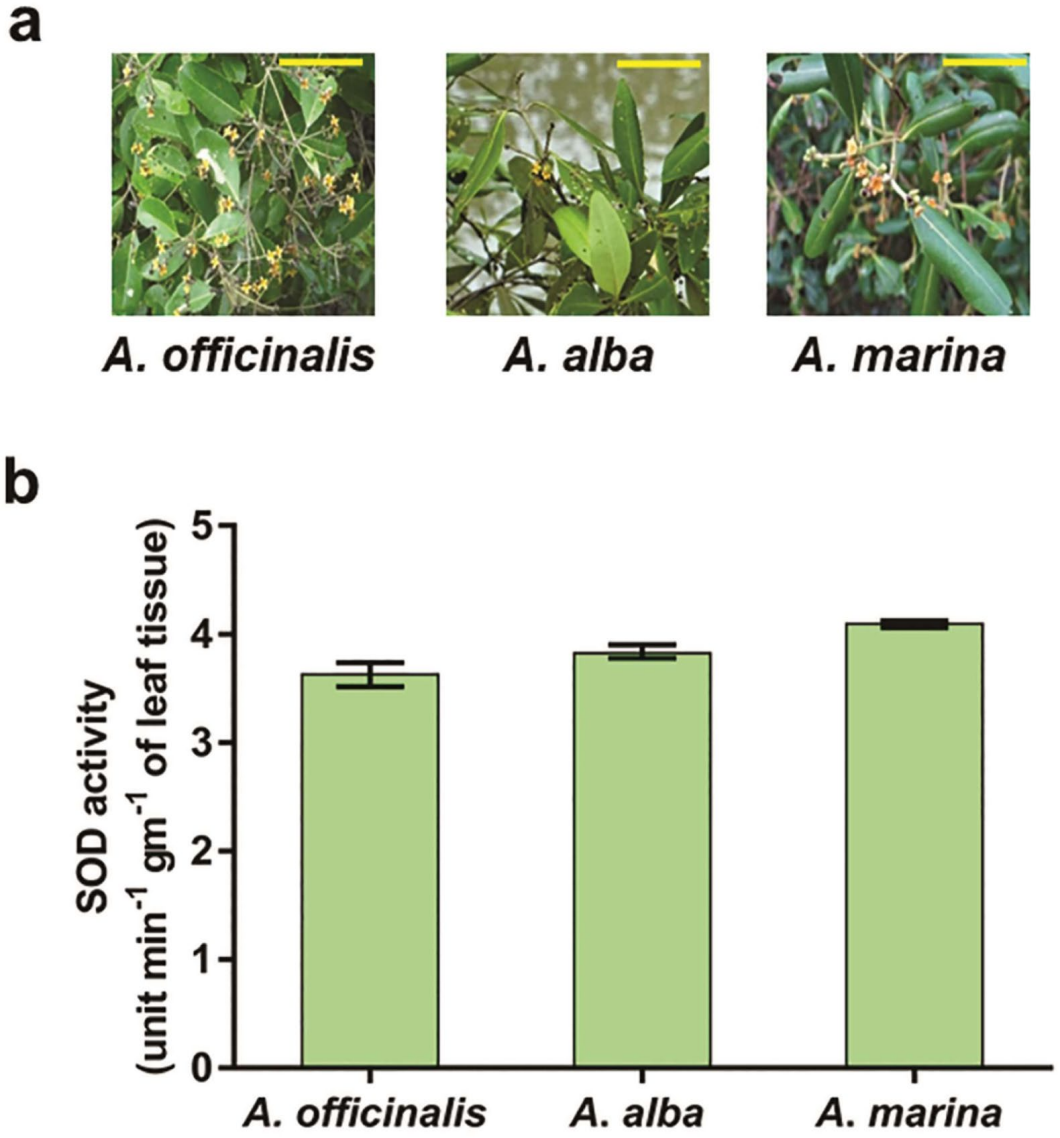
*A*. *marina* displayed highest SOD activity among 3 species. (**a**) Photograph of twigs with inflorescence of 3 species of *Avicennia* collected from Sundarban mangrove. Horizontal yellow bar represents 10 cm. (**b**) SOD activity assay from leaf extract of these 3 species. SOD activity is expressed as unit of enzyme present in each gram of leaf tissue converting the substrate into product in a minute plotted in y-axis. Each bar graph represents the mean of 6 biological replicates collected from 6 different locations (n = 6) and error bar as SD.

The present study started with screening out the particular *Avicennia* species with maximum SOD activity. The free radical scavenging activity of 3 different species of *Avicennia* from the Indian mangrove forest (Figure 1a) was compared in terms of the SOD activity of the leaf. The comparative SOD activity in the crude extract (in unit per minute) prepared from each gram of leaf tissue of these 3 species is shown in Figure 1b in which *A*. *marina* displayed the highest activity among all the 3 species. For each species, leaf tissues were collected from 6 different populations grown at 6 different locations of the mangrove forest. The intra-species variation in SOD activity was very insignificant as evident from the standard deviation of the data that ruled out the possibilities of experimental error while performing the enzyme assay as well as the impact of variation in environmental factors during sample collection.

### A 16 kDa protein of *A. marina* displayed SOD activity

**Figure 2:**
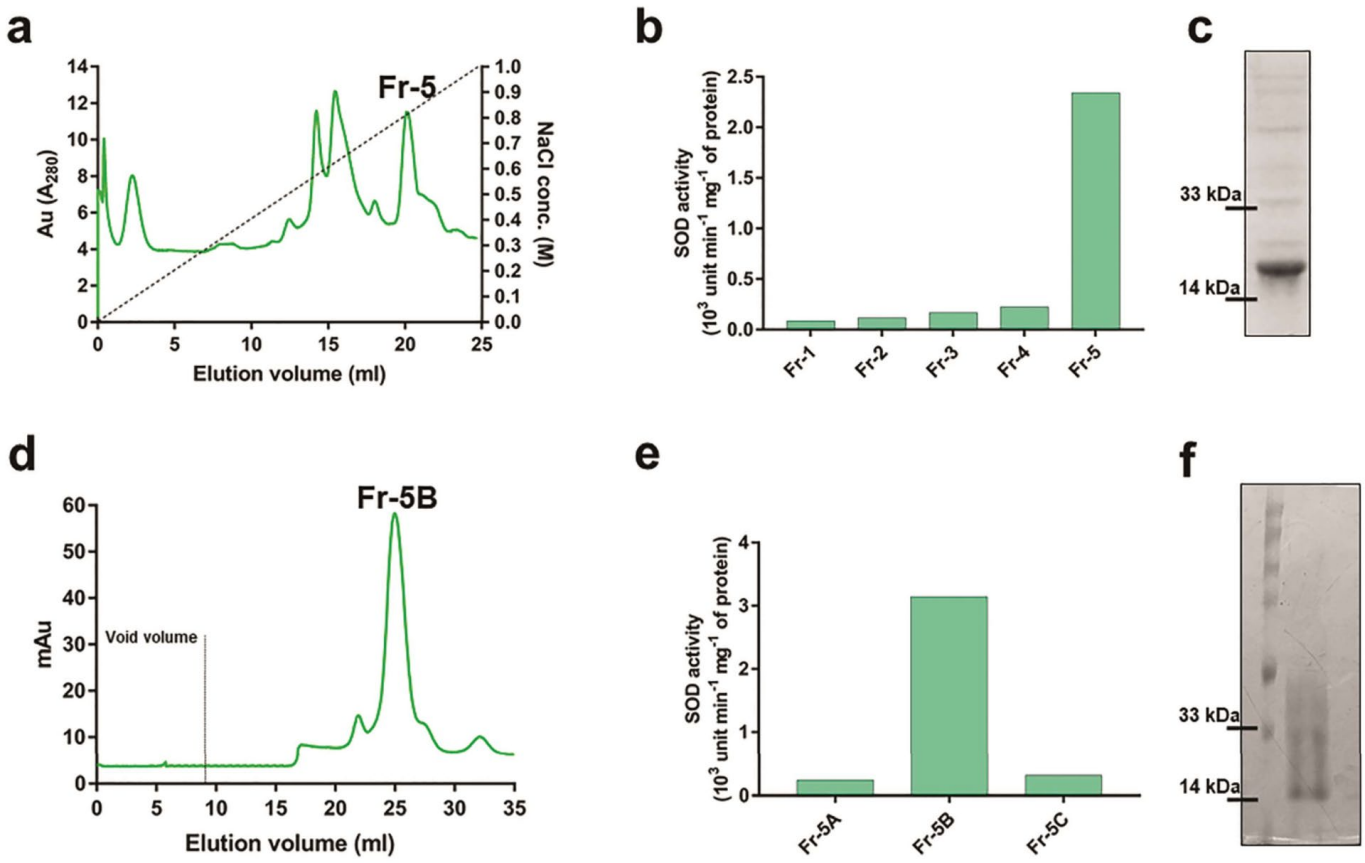
Purification of *Am*_SOD from *A*. *marina* leaf. Supernatant fraction after 60% ammonium sulfate cut of leaf extract was fractionated in anion exchange column followed by gel filtration. Chromatograms are shown in (**a**) and (**d**). The A_280_ of eluted fractions are in y-axis versus elution volume in x-axis. Void volume (9.2 ml) of the gel filtration column is demarcated by dashed line in (**d**). SOD activity assay (in y-axis) was done to screen for the presence of *Am*_SOD in each column eluted fraction (in x-axis) as shown in (**b**). Fraction number 5 (Fr-5) of anion exchange chromatography showed highest SOD activity and partially purified *Am*_SOD protein in SDS-PAGE (**c**). Fr-5 was re-fractionated in gel filtration column and Fr-5B displayed highest SOD activity (**e**) with purified *Am*_SOD in > 85% homogeneity in SDS-PAGE (**f**).

Next, by employing a three-step purification strategy, the native SOD enzyme was purified from *A*. *marina* leaf into partial homogeneity. In step-1, proteins present in the pellet fraction after 60% ammonium sulfate cut were obtained and were subjected to strong anion exchange chromatography in step-2. Five eluted fractions obtained from step-2 (Figure 2a) were screened by SOD enzyme assay and fraction 5 displayed the highest SOD activity (Figure 2b). In the SDS-PAGE profile of this fraction, a 16 kDa protein was found to have been enriched along with some other proteins (Figure 2c). Hence, fraction 5 was re-fractionated in a gel filtration column in step-3. In gel- filtration, fraction 5 was separated into 3 sub-fractions (Fr 5A to 5C in Figure 2d). Fraction 5B was found to have the highest SOD activity (Figure 2e) and contain a 16 kDa protein with >90% purity in SDS-PAGE (Figure 2f). This protein band was excised from the gel, trypsin-digested, and analyzed by LC-MS/MS. As shown in supplementary Table S1, a total of 6 unique peptides were identified from this 16 kDa protein and all of them showed a significant match with a Cu, Zn-superoxide dismutase of *A*. *marina* in the UniProt database (Acc. no. Q9AXH2). Together, these 6 peptides account for about 48% sequence coverage to the intact protein. This identified protein is designated as *Am*_SOD throughout the entire study.

### Recombinant *Am*_SOD was homodimeric

**Figure 3:**
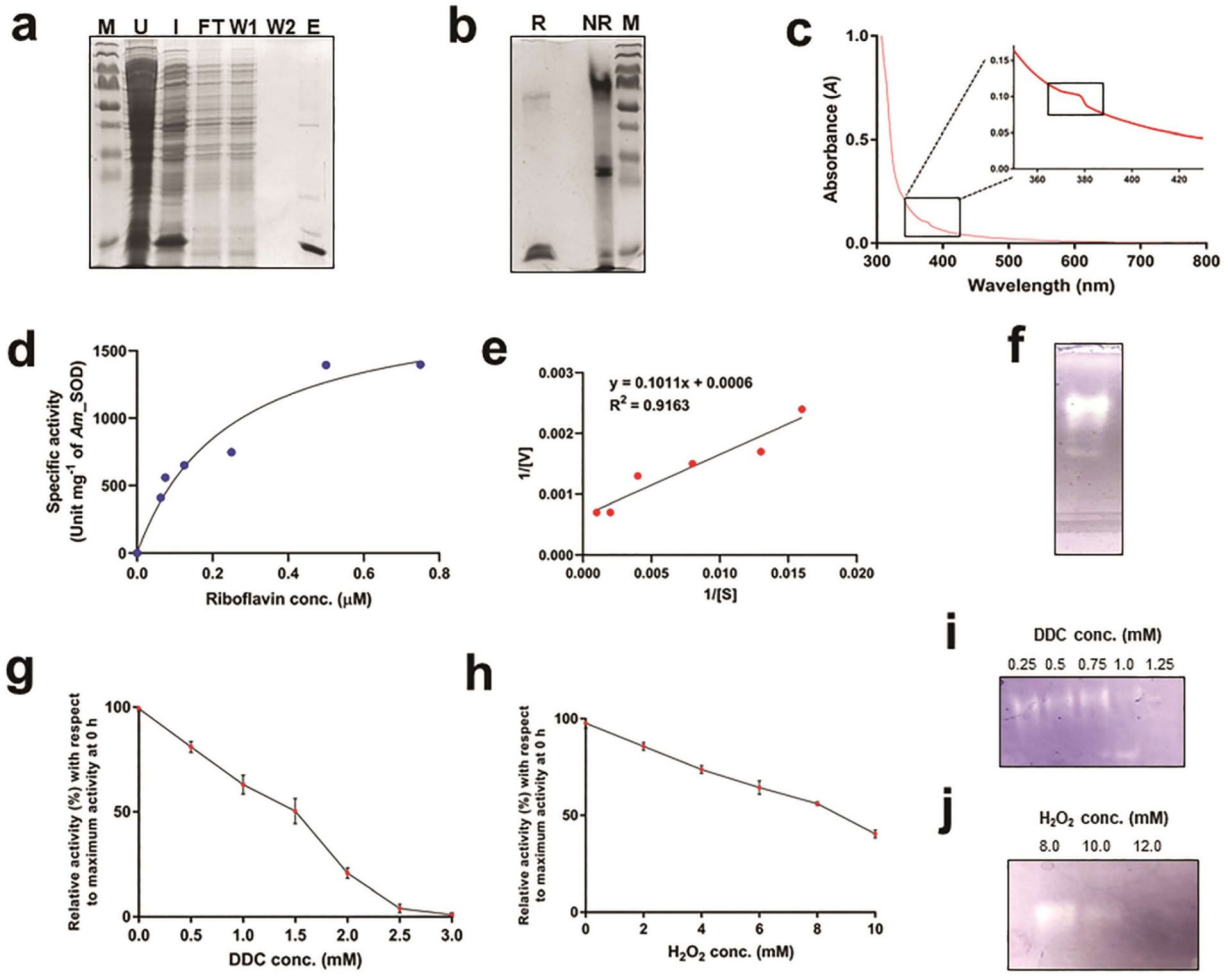
Recombinant *Am*_SOD is a functionally active superoxide dismutase. (**a**) 6xHis-tagged *Am*_SOD was purified by Ni–NTA chromatography in soluble form. Lane U; uninduced control, lane I; supernatant fraction after sonication of IPTG-induced *E*. *coli* cells harboring *Am*_SOD-pET22b+ construct. Flow-through (lane FT) from supernatant fraction after binding with Ni–NTA. Beads were washed twice (lanes W1 and W2) with 40 mM imidazole. Column-bound *Am*_SOD was eluted (lane E) with 250 mM imidazole. Left margin (M) is MW marker. (**b**) 12% SDS-PAGE showing *Am*_SOD dimer (~ 32 kDa) under non-reducing condition without β-ME (lane NR) and monomer (~ 16 kDa) under reducing condition with β-ME (lane R). Right margin (M) is MW marker. (**c**) Absorption spectra of 0.8 mg/ml of *Am*_SOD at 300–800 nm (in x-axis) wavelengths showing an electronic transition at 380–400 nm region which is magnified and shown in inset. Electronic transition suggests an interaction of Cu^2+^ with imidazole ring of His-62. (**d**) Michaelis–Menten kinetics curve showing specific activity of *Am*_SOD (0.005 mg/ml) expressed as unit of enzyme per milligram of protein (in y-axis) as a function of riboflavin concentrations (µM, in x-axis). (**e**) Lineweaver–Burk plot showing linear regression of *Am*_SOD kinetics. [V] and [S] are reaction velocity (y-axis) and substrate concentrations (x-axis) respectively. Linear relationship (R^2^) and straight-line equation of the plot are shown. V_max_ and K_m_ were determined as inverse of y- and x-intercepts respectively. (**f**) Zymography in native PAGE showing SOD activity where 1 µg *Am*_SOD formed a hyaline zone and rest of the gel turned blue due to NBT oxidation by superoxide radicals generated from riboflavin. Dose- dependent inhibition of specific activity of 0.005 mg/ml of *Am*_SOD by increasing concentrations (in x-axes) of Sodium diethyldithiocarbamate trihydrate/DDC (**g**) and hydrogen peroxide/H_2_O_2_ (**h**). The SOD activity is presented here as a percentage of activity (in y-axis) at a certain inhibitor concentration after a 0.5 h of incubation with respect to the maximum activity at 0 h at that particular inhibitor concentration. (**i**) and (**j**) showing zymography of dose-dependent inhibition of 1 µg of *Am*_SOD activity incubated with increasing concentrations of DDC and H_2_O_2_ respectively.

The mass-spectrometry deduced sequence of the purified protein with SOD activity was used to identify the gene from *A. marina* genome (NCBI genome ID: 16351)^25^. tBLASTn search with *Am*_SOD amino acid sequence revealed a 768 bp long mRNA transcript (GeneBank Acc. AF328859.1). The transcript was found to contain a 459 bp long ORF coding for the full-length *Am*_SOD protein. The full-length mRNA transcript and the ORF were separately PCR-amplified from the first-strand cDNA prepared from the total RNA of *A. marina* leaf. The full-length *Am*_SOD gene (with exons and introns) was also PCR amplified from the genomic DNA. The 768 bp long mRNA transcript and the full-length gene were separately cloned in the pCR^TM^2.1 vector by the TA-cloning method. The ORF was cloned in Nde1 and Xho1 sites of the pET22b+ vector under the control of the T7 promoter. Sequence analysis and comparison of these 3 cloned inserts revealed that the 2027 bp long gene consists of 7 exons with 6 introns. Upon splicing, a 768 bp long mRNA transcript is generated which consists of 459 bp long ORF with a 46 bp long 5′ UTR and a 263 bp long 3′ UTR. The domain architecture and nucleotide sequence of the full-length *Am*_SOD gene are shown in supplementary Fig. S1a, b. A positive clone with the *Am*_SOD cDNA insert positioned in an accurate reading frame (supplementary Fig. S2) was selected for recombinant expression. The expression of N-terminal 6xHis tagged recombinant *Am*_SOD was induced in *E. coli* cells with IPTG and the recombinant protein was found to be in a soluble form. The recombinant *Am*_SOD was then purified using Ni-NTA affinity column under native condition (Figure 3a) followed by the second round of purification in size exclusion column to remove undesired aggregates and non-specific *E. coli* proteins. The yield of recombinant *Am*_SOD was ~8–10 mg L^−1^ of culture. The oligomerization status of the purified Am_SOD was checked in SDS-PAGE shown in Figure 3b. In non-reducing SDS-PAGE, *Am*_SOD appeared at ~33 kDa region, which corresponds to the MW of a dimer. However, in presence of β-mercaptoethanol, only the monomeric form was visible on the gel.

### *Am*_SOD displayed characteristic Cu, Zn-superoxide dismutase activity

Sequence analysis of *Am*_SOD revealed the presence of a conserved SOD catalytic domain with multiple copper and zinc ion binding sites. The spectral pattern of *Am*_SOD in the visible region (300–800 nm) showed the evidence of electronic transitions at a region between 380–400 nm indicative of Cu^2+^ interaction with the imidazole ring of Histidine-62 (Figure 3c). Therefore, the enzymatic activity of the purified *Am*_SOD was studied by performing an assay using the riboflavin-NBT system. The principle of this assay is based on the fact that illumination of riboflavin generates free superoxide radicals which can convert NBT into blue-colored formazan which is spectrophotometrically measured at 560 nm. However, in presence of SOD, these radicals are scavenged and hence, the NBT conversion is reduced. Therefore, the high the A_560_ value the less the amount of SOD enzyme present in the system and vice versa. As shown in Figure 3d, *Am*_SOD followed a typical Michaelis–Menten kinetics with an increasing concentration of riboflavin as substrate. The kinetic data were then plotted in a double reciprocal Lineweaver–Burk plot shown in Figure 3e. The V_max_ and K_m_ of recombinant *A*m_SOD were obtained to be 1557.14 unit/mg and 0.15738 µM respectively in 50 mM Tris buffer at pH 7.5. In addition to spectrophotometric assay, an in-gel activity assay was also performed in native PAGE shown in Figure 3f in which the *Am*_SOD appeared as a hyaline zone while the rest of the gel turned blue due to the oxidation of NBT. The enzymatic activity of Cu, Zn*-*SOD is specifically inhibited by diethyldithiocarbamate (DDC) and H_2_O_2_. In this study, the activity of *Am*_SOD was specifically inhibited in a dose-dependent manner by sodium diethyldithiocarbamate trihydrate, and the IC_50_ was obtained at 1.5 mM (Figure 3g). As compared to DDC, H_2_O_2_ was found to have a less inhibitory effect on *Am*_SOD as the IC_50_ value was obtained at 8 mM (Figure 3h). A similar pattern of *Am*_SOD inhibition was observed in zymography assay performed with DDC and H_2_O_2_ (Figure 3i, j). The activity of *Am*_SOD was fully inhibited by both inhibitors at high concentrations.

### *Am*_SOD showed halotolerance

**Figure 4:**
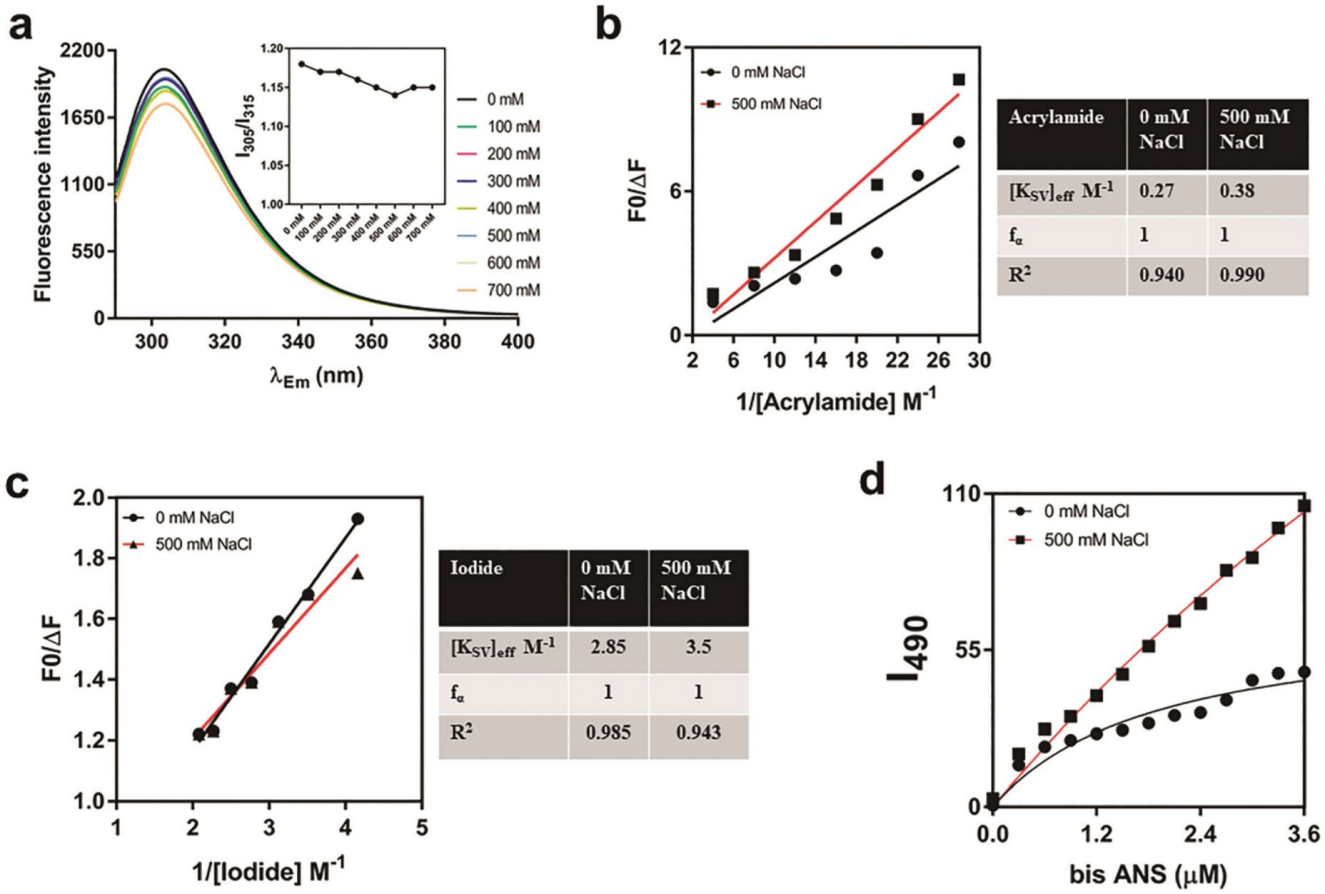
Studying salt tolerance of *Am*_SOD by fluorescence spectrometry. (**a**) Fluorescence spectra showing tyrosine autofluorescence intensity (in y-axis) of 0.05 mg/ml of *Am*_SOD incubated with various NaCl concentrations and scanned at emission wavelength from 290 to 400 nm (in x-axis). A plot in the inset showing no noticeable change in normalized fluorescence intensity of *Am*_SOD (ratio between intensities at 305 to 315; in y-axis) at increasing NaCl concentration (in x-axis). (**b**) and (**c**) showing modified Stern–Volmer plots of tyrosine fluorescence quenching of *Am*_SOD by increasing concentrations (in x-axis) of acryalmide and iodide respectively. In each quenching experiment, *Am*_SOD was incubated with 500 mM NaCl or without salt treatment (control). Fluorescence quenching data is presented here on y-axis as a ratio between fluorescence intensity without quencher (F0) and difference in fluorescence intensity after adding quencher (ΔF). The calculated values of quenching parameters are displayed in the tables adjacent to each Stern–Volmer plot. (**d**) Plot showing surface hydrophobicity in terms of fluorescence emission spectra at 490 nm (in y-axis) of *c* treated with or without NaCl and then titrated with increasing concentrations (in x-axis) of Bis-ANS.

**Figure 5:**
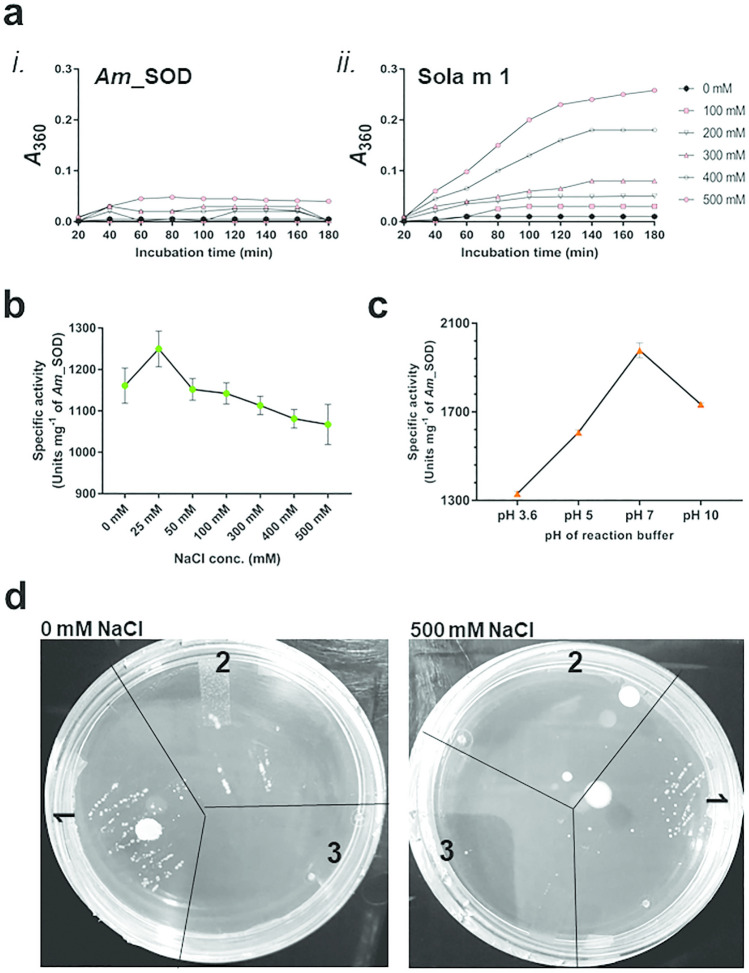
Impact of salt and pH on *Am*_SOD activity. (**a**) Plot of single light scattering experiment showing resistance of *Am*_SOD (*i*) to NaCl-induced aggregation. No significant increase in absorbance of salt-treated *versus* untreated *Am*_SOD at 360 nm (in y-axis) was observed over time (in x-axis). A salt-sensitive profilin protein, Sola m 1 from eggplant (*ii*) was used as control. (**b**) and (**c**) Plots showing specific activity (in y-axis) of 0.005 mg/ml of *Am*_SOD in presence of increasing concentrations of NaCl and 4 different pH values (in x-axes) respectively. 1.17 µM riboflavin was used as substrate in all reactions. Each data point is a mean of triplicate measure and SD as error bars. (**d**) Functional complementation of *Am*_SOD in *E. coli* on LB-agar plates with 500 mM NaCl (salt treated) and with 0 mM salt (untreated control). Both the plates were supplemented with methyl viologen to induce oxidative stress, IPTG for protein induction, and ampicilin for selection. Appearance of growth was observed for *sod* double mutant strain QC774 transformed with *Am*_SOD construct (area 1) on both the plates suggesting the ability of *Am*_SOD to remain functionally active for combating oxidative stress under high saline condition. K12 strain with functional native *sod* genes harboring pET22b+ vector (area 2) grew only in zero salt plate (under oxidative stress only). QC774 strain harboring pET22b+ vector (area 3) failed to grow under oxidative as well as salinity stress.

Having a mangrove origin, *Am*_SOD was expected to be a salt-tolerant protein. The salt tolerance was investigated and established through a couple of experiments. First, the tyrosine (Tyr)-fluorescence spectra of *Am*_SOD were investigated at various NaCl concentrations (Figure 4a). In absence of salt, the wavelength of maximum emission for *Am*_SOD was obtained at 310 nm, which is typical of a tyrosine residue. No significant change in the Tyr-fluorescence of Am_SOD was observed in presence of NaCl at a concentration as high as 700 mM. Next, to understand further the salt-tolerant feature of *Am*_SOD we performed the Tyr-fluorescence quenching experiment using acrylamide and potassium iodide (KI) as 2 complementary sets of water-soluble quenchers. Acrylamide is a neutral quencher and can enter the interior of a protein. On the other hand, iodide is a negatively charged and bulky quencher that can quench the fluorescence of the surface residues. *Am*_SOD has no tryptophan residue but only a single tyrosine residue in its sequence. Therefore, in this study, the quenching data were analyzed by Stern–Volmer plot considering a homogenous emission from a single tyrosine. The quenching constant of this single tyrosine is reported here as effective Stern–Volmer constant (K_SV_)_eff_. The acrylamide and KI quenching data of *Am*_SOD under control and high NaCl stress are represented in Figure 4b, c respectively. The values of (K_SV_)_eff_ and f_α_ (quenchable fraction) are displayed in the tables adjacent to each corresponding plot. Considering the presence of only one tyrosine in Am_SOD, 100% quenching of fluorophore was observed in both experiments. Hence, this tyrosine residue is presumably located on the surface of *Am*_SOD. In acrylamide and KI quenching, insignificant change in the (K_SV_)_eff_ of *Am*_SOD was observed both in absence of NaCl as well as in presence of 500 mM NaCl. The data indicated that there was a marginal conformational change in *Am*_SOD in presence of a high concentration of NaCl as compared to no salt control. The conformational behavior of *Am*_SOD in presence of NaCl was further investigated by Bis-ANS fluorescence assay (Figure 4d) that exploits the surface hydrophobicity of a protein. Bis-ANS is a conformation-sensitive hydrophobic probe with a low quantum yield. However, it becomes highly fluorescent when binds to the hydrophobic pockets exposed on the protein surface. Unlike salt-sensitive proteins where hydrophobic pockets get buried under salt stress, *Am*_SOD displayed a significant increase (~50%) in surface hydrophobicity. Next, the salt-induced aggregation pattern of *Am*_SOD was studied by a single light scattering experiment shown in Figure 5a-i. A previously reported salt-sensitive and allergenic profilin Sola m 1 (a gift from Dr. Swati Gupta Bhattacharya of Bose Institute, Kolkata, India) isolated from eggplant^26^ was used as a control to compare the results. *Am*_SOD did not show any aggregation even in the presence of 500 mM NaCl as evident from very insignificant/no increase in the absorbance at 360 nm. On the contrary, Sola m 1 started forming aggregates in the presence of 400 mM NaCl (Figure 5a-ii). All the above experiments are focused on studying the salt-induced conformational and structural changes in *Am*_SOD. In addition to these, the impact of salt concentration on the catalytic activity of *A*m_SOD was investigated as shown in Figure 5b. *Am*_SOD exhibited catalytic activity in presence of a wide range of NaCl concentrations. Maximum activity was observed at 25 mM NaCl and a further increase in salt concentration resulted in a gradual decrease in the specific activity. However, the enzymatic activity of *Am*_SOD was not drastically altered (<25% reduction) in presence of NaCl as high as 250 mM as compared to no salt control. Altogether, it was found that the biological function of *Am*_SOD was not considerably affected by high salt stress.

### *Am*_SOD displayed enzyme activity at alkaline pH

The effect of pH on recombinant *Am*_SOD activity was studied using buffer systems of 4 different pH values. As illustrated in Figure 5c, the SOD activity was almost diminished at acidic pH of 3.6. However, the protein displayed enzyme activity in mildly acidic pH and the pH optimum was obtained at pH 7, which is a physiological pH. Interestingly, considerable retention of enzyme activity of *Am*_SOD was observed at a strongly alkaline pH of 10.

### Functional complementation of salt tolerant *Am*_SOD in *E. coli*

For functional identification of *Am*_SOD gene, a genetic complementation test was performed in a double SOD deficient mutant strain (Δ*sod*A and Δ*sod*B) of *E*. *coli* named QC774. The cells were transformed with *Am*_SOD cloned in pET22b+ vector. For control, QC774 cells and wild type *E. coli* K12 strain transformed with pET22b+ vector without any insert were used. Transformed cells were first selected on LB-agar plates containing ampicilin. An individual transformed colony was then streaked on LB-agar plate supplemented with ampicilin, methyl viologen dichloride for inducing oxidative stress, and 500 mM NaCl for inducing salt stress. For untreated control, LB-agar plate was used without NaCl but with ampicilin and methyl viologen. Protein expression was induced by adding IPTG in all the plates. As shown in Fig. 5d, only QC774 cells harboring *Am*_SOD constructs were able to grow under oxidative stress as well as salinity stress. On contrary, K12 cells with functional *sod* genes were able to survive only under the oxidative stress but couldn’t grow in presence of high NaCl concentration. QC774 cells harboring empty vector were unable to survive under oxidative as well as salinity stress. This observation suggests the salt-tolerant feature of *Am*_SOD in addition to its potential role in combating oxidative stress.

### *Am*_SOD displayed a certain degree of heat tolerance

**Figure 6:**
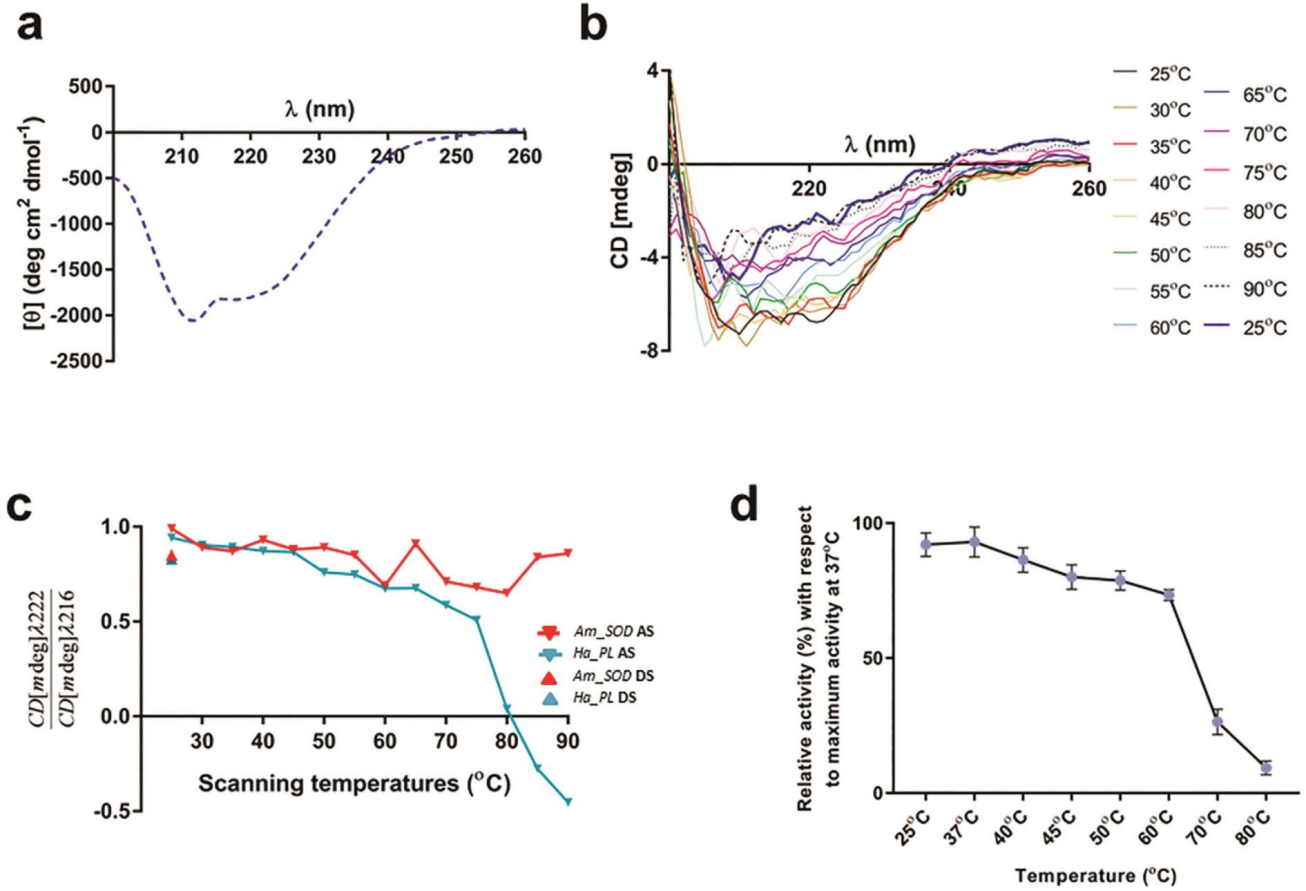
*Am*_SOD displayed certain degree of heat tolerance. (**a**) CD spectra showing molar ellipticity (in x-axis) of 5 µM of *Am*_SOD at wavelengths 200–260 nm (in x-axis) and at 25 °C. (**b**) Step-scan showing raw CD millidegrees (in y-axis) of 5 µM of *Am*_SOD at wavelengths 200–260 nm (in x-axis) within a temperature range from 25 to 90 °C with a 5 °C increment. After 90 °C, CD spectra were recorded once again at 25 °C. (**c**) Melting curve of *Am*_SOD showing no noticeable change in the fractions of α-helices and β-sheets present in the protein (ratio of CD millidegree at 222 and 216 nm, in y-axis) in an ascending scan or AS (step-wise from 25 to 90 °C) as well as in a descending scan or DS (direct from 90 to 25 °C). Melting curve of sunflower pectate lyase Hel a 6 (Ha_PL), a heat-sensitive control protein is also plotted for comparison. Ha_PL showed reversible thermal denaturation in which the protein was fully unfolded at 90 °C but partially refolded upon cooling. (**d**) Effect of temperature (in x-axis) on specific activity of 0.005 mg/ml of *Am*_SOD. The SOD activity is presented here as a percentage of activity (in y-axis) at a certain temperature with respect to the SOD activity at 37 °C (considered as optimum activity). 1.17 µM riboflavin was used as substrate in each reaction. Each data point is a mean of triplicate measure and SD as error bars.

Deconvolution of CD spectra of *Am*_SOD (Figure 6a) at 25 °C revealed a correctly folded protein with predominantly β-sheets as evident from the minimum obtained at 215 nm. Also, a characteristic shoulder at 222 nm indicated the presence of a certain degree of α-helices. In step-wise thermal scanning, *Am*_SOD did not exhibit temperature-dependent denaturation since an inconspicuous change in the CD signal was observed at 90 °C as compared to what was observed at 25 °C (Figure 6b). A melting curve of *Am*_SOD shown in Figure 6c represents the ratio between α-helical fraction and β-sheeted fractions present in this protein at various temperatures. No significant decline in this melting curve of *Am*_SOD was observed when the temperature was gradually raised from 25 to 90 °C indicating no heat-induced conformational change in the protein. For comparison, a previously reported heat-sensitive pectate lyase of sunflower designated as Hel a 6 (a gift from Dr. Nandini Ghosh of Vidyasagar University, West Bengal, India) was used as a control. Hel a 6 protein was reported to show reversible heat denaturation^27^. Hence, the Hel a 6 melting curve exhibited a sharp decline with increasing temperature (AS or ascending scan) and the native folds were gradually lost. However, Hel a 6 partially refolded from a fully denatured state when the CD-scanning temperature was set back to 25 °C. To substantiate this observation, the effect of temperature on the catalytic activity of *Am*_SOD was investigated as shown in Figure 6d. Unlike the conformation-dependent melting curve in Figure 6c, the enzymatic activity of *Am*_SOD remarkably declined at temperatures as high as 70 °C and 80 °C. However, *Am*_SOD was able to retain up to 70% of its catalytic activity at 60 °C.

### Reduction in surface hydrophobicity is linked to halotolerance of *Am*_SOD

**Figure 7:**
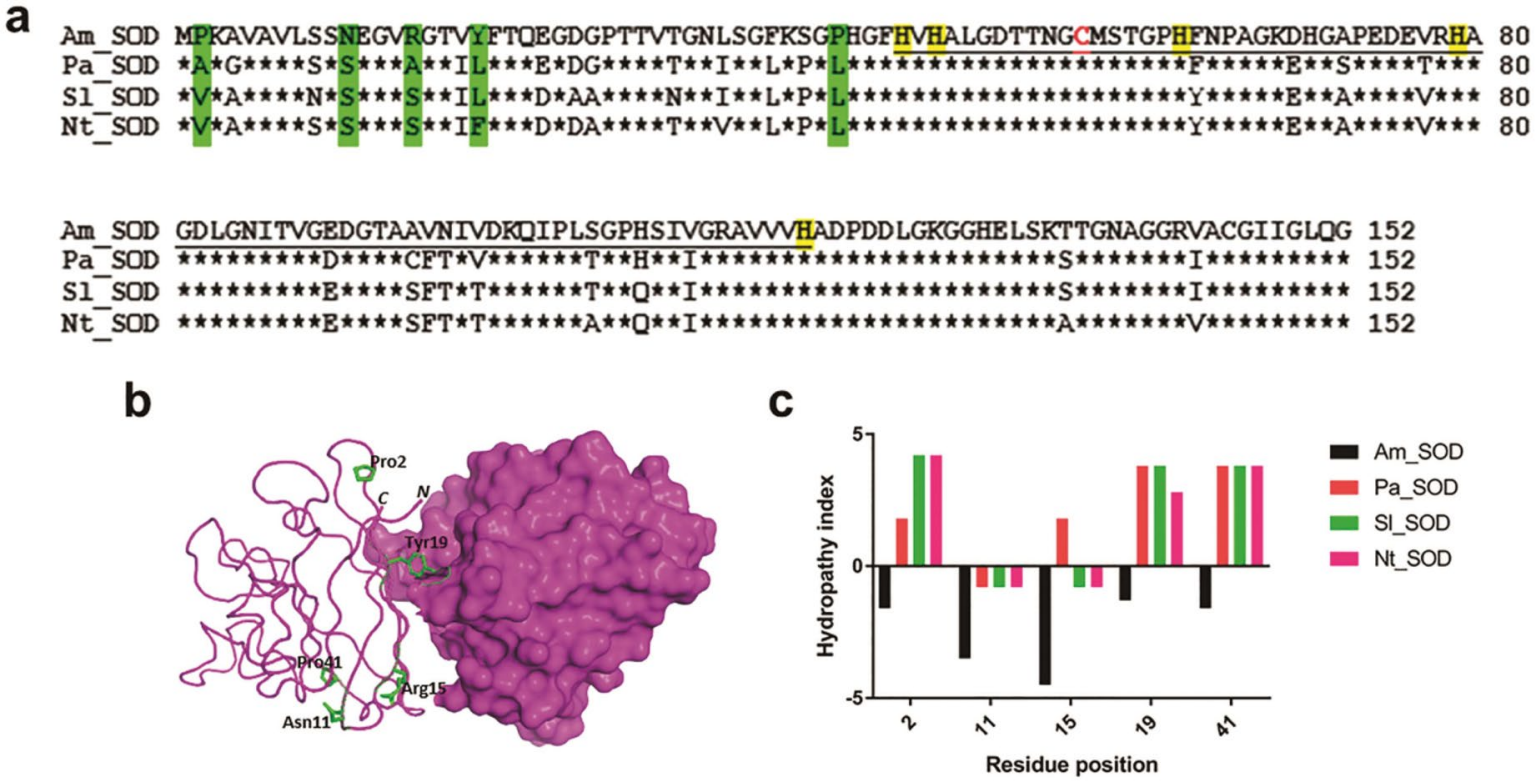
Mapping of critical residues conferring salt tolerance to *Am*_SOD by in silico studies. (**a**) Multiple sequence alignment of amino acid sequences of *Am*_SOD with 3 non-halophilic Cu, Zn-SOD proteins from *Potentilla* (Pa_SOD), tomato (Sl_SOD), and tobacco (Nt_SOD) using Clastal Omega server (https://www.ebi.ac.uk/Tools/msa/clustalo/). Identical residues on aligned sequences are shown in asterisks. 5 N-terminal residues in *Am*_SOD with decreased hydrophobicity as compared to the non-halophilic counterparts are highlighted in green. The highly conserved catalytic domain is underlined. The cysteine residue responsible for dimer formation is shown in red and the histidine residues responsible for metal ligand (Cu and Zn) binding are shown in yellow. (**b**) The homology model of *Am*_SOD dimer built in SWISS-MODEL server (https://swissmodel.expasy.org/) shown as cartoon and surface representation for chain-A and chain-B respectively using PyMol v2.5 (https://pymol.org/2/). Atomic structure of those 5 critical residues is labeled on chain-A. (**c**) Plot showing changes in hydropathy index values (in y-axis) in these 5 critical residues of *Am*_SOD as compared to corresponding residues in the same position (in x-axis) on 3 non-halophilic Cu, Zn-SOD proteins.

**Figure 8:**
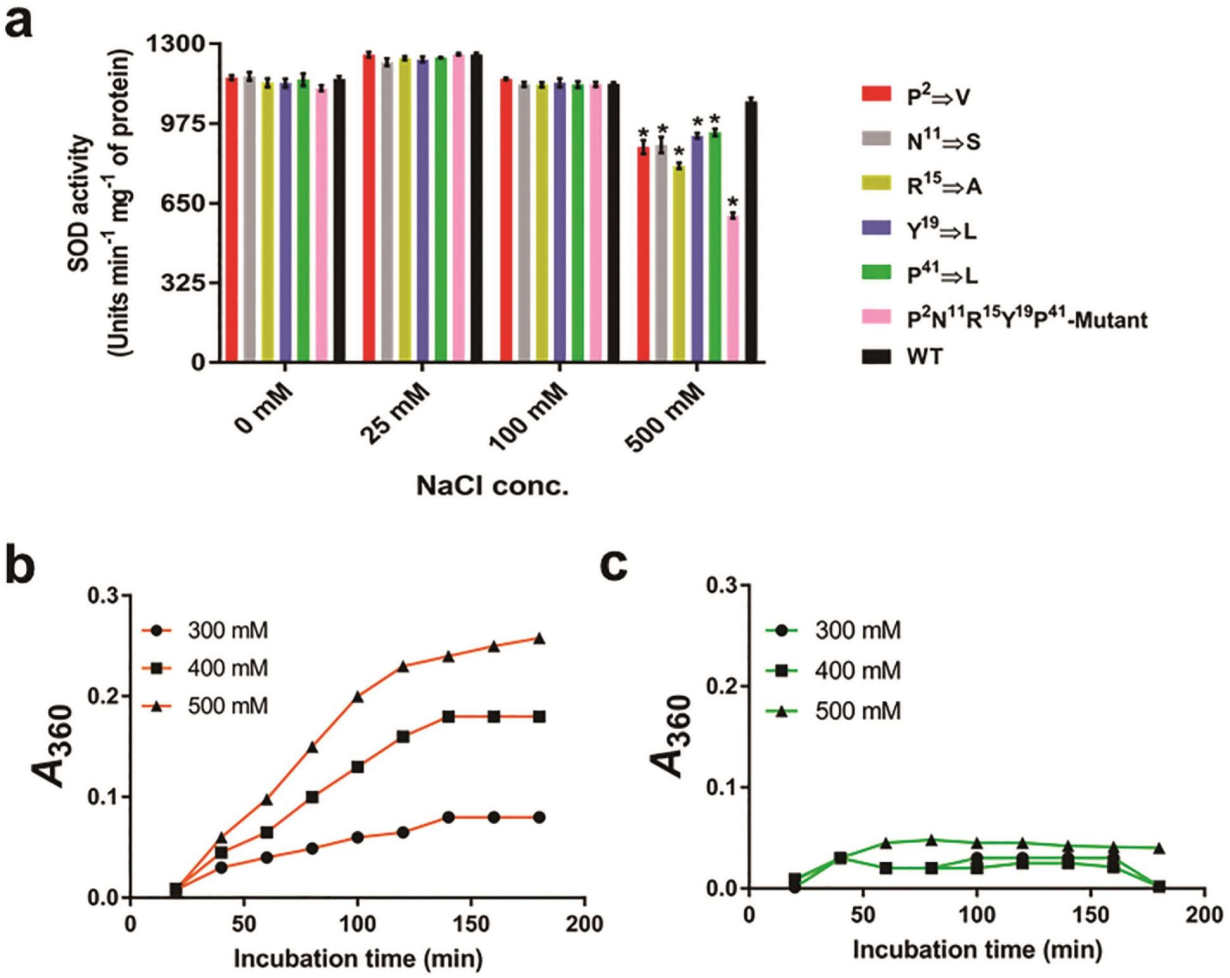
Mutation in 5 N-terminal critical residues resulted in decrease in salt tolerance of *Am*_SOD. (**a**) Plot showing specific activity (in y-axis) of wild type/WT *Am*_SOD and 6 mutant versions under various NaCl concentrations (in x-axis). Significant reduction (*p* < 0.05 as asterisk) in SOD activity was observed in all the 6 mutants at 500 mM NaCl where the maximum reduction (~ 54%) was observed for the multiple-point mutant harboring all the 5 point mutations. (**b**) and (**c**) Plots of single light scattering experiment showing NaCl-induced aggregation of the multiple-point mutant *versus* resistance to NaCl-induced aggregation of *Am*_SOD respectively. Here, 3 different NaCl concentrations were used to treat the proteins and absorbance at 360 nm (in y-axis) was scanned at different time points (in x-axis).

A rational mutagenesis approach was undertaken to understand the role of a few selected residues in conferring salt tolerance to *Am*_SOD. Previous studies have shown that increased salt tolerance of a halophilic protein is associated with a noticeable increase in surface-exposed charge residues (negatively charged in particular) and reduction in surface hydrophobicity^28–30^. In this study, a comparison of *Am*_SOD with 3 non-halophilic Cu, Zn-SODs (Pa_SOD from *Potentilla*, Nt_SOD from tobacco, and Sl_SOD from tomato) by multiple sequence alignment (Figure 7a) revealed the presence of 8 less-hydrophobic residues in the N-terminus as compared to more hydrophobic residues on the corresponding positions of non-halophilic SODs. However, no significant change in surface-exposed charged residues was observed between *Am*_SOD and non-halophilic SODs. Hence, we anticipated the involvement of these 8 residues in the salt tolerance of *Am*_SOD. Out of 8, 5 residues were found to be sufficiently surface exposed on the tertiary structural model of *Am*_SOD (Figure 7b) and were estimated to have high SASA values as listed in supplementary Table S2. Residues of non-halophilic SODs corresponding to these 5 residues were also found to be solvent accessible. Each of these 5 residues on *Am*_SOD was found to have the lowest hydropathy index value (i.e. lowest hydrophobicity) as compared to the corresponding residues on 3 non-halophilic SODs (Figure 7c). Here, we decided to replace each of these 5 residues on *Am*_SOD with the residue having the highest hydropathy index value on the corresponding position among the 3 non-halophilic SODs. The strategy of amino acid substitution is illustrated in supplementary Table S2. In this way, 5 single-point mutants were generated by site-directed mutagenesis. A sixth mutant carrying all the 5 substitutions in the same protein was also generated by gene synthesis. The recombinant versions of all these 6 mutants were expressed in soluble forms and were found to remain in dimer as shown in non- reducing SDS-PAGE (data not shown). Now, the superoxide dismutase activity of these mutants was compared to that of the WT *Am*_SOD in gradually increasing NaCl concentrations. As shown in Fig 8A, the SOD activity of all the 6 mutants was nearly similar to that of the WT enzyme when assayed in presence of 25, and 100 mM NaCl. However, a significant reduction (*p*<0.05) in SOD activity of the 6 mutants was noticed when the NaCl concentration was increased up to 500 mM. Among the 6 mutants, the multiple-point mutant displayed maximum reduction (>50%) in SOD activity indicating a cumulative impact of these 5 substitutions on increasing the surface hydrophobicity and subsequently perturbing the halotolerance of the protein. In another experiment, the single light scattering pattern of the multiple-point mutant was compared with that of the WT *Am*_SOD under high salt stress. As shown in Figure 8b, the multiple-point mutant exhibited salt-induced aggregation in presence of 500 mM NaCl as compared to WT *Am*_SOD (Figure 8c) that remained considerably soluble.

## Discussion

The present study presents a comprehensive characterization of a novel SOD enzyme isolated from a mangrove species of Indian origin using biochemical and biophysical methods. Mangroves are adapted to survive in high salinity environments. The generation of free radicals in the form of reactive oxygen species is a major manifestation of salt stress. To combat this challenge, mangroves are equipped with strong antioxidant systems that can function in a highly saline microenvironment. SOD enzymes are crucial members of the enzymatic antioxidant system. In this study, a high SOD activity of *A*. *marina* among 3 different *Avicennia* species was found to be associated with a 16 kDa protein designated as Am_SOD. The purity level and yield of natural *Am*_SOD protein purified from *A*. *marina* leaf were found to be considerably low. Hence, the full-length gene coding for this protein was isolated and purified in recombinant form. The analysis of the *Am*_SOD sequence revealed the presence of a conserved domain along with 6 conserved histidine residues responsible for metal ion (Cu^2+^ and Zn^2+^) binding which are characteristic of a Cu, Zn-SOD. Any organelle-specific putative signal peptide was not found in *Am*_SOD and its sequence showed homology mostly with cytosolic SOD enzymes. Interestingly, *Am*_SOD was sensitive to H_2_O_2_, a potent inhibitor of various Cu, Zn-SODs^31^. Usually, peroxisomal SODs have been reported to have less sensitivity for H_2_O_2_ as observed in a recently characterized Cu, Zn-SODs from rice^32^. Hence, the possibility of the peroxisomal location of *Am*_SOD can be ruled out. A further in situ localization study is warranted to confirm the subcellular location of this protein. Recombinant *Am*_SOD was found to be a functional enzyme since it retained all the native folds as well as the catalytic activity. The kinetic data of *Am*_SOD represents a high substrate affinity and strong superoxide dismutation activity as compared to many previously reported Cu, Zn-SODs of eukaryotic origin. Such a robust activity of *Am*_SOD is thought to be the key for homeostasis of the exceptionally high level of ROS resulting from salinity stress and thereby protecting the cellular components from oxidative damage. To perform the biological activity, *Am*_SOD is thought to remain functional in a stressful microenvironment with extreme physiological conditions like high osmolarity and ionic strength. Here, we established the halotolerant feature of *Am*_SOD in terms of conformational stability and resistance to aggregation under high salt stress. The conformational stability of *Am*_SOD as observed in its tyrosine fluorescence quenching pattern was similar to the tryptophan fluorescence quenching reported for a halophytic rice protein PINO1^33^. In Bis-ANS spectrofluorometric assay, a considerable increase in surface-exposed hydrophobic pockets in presence of high NaCl concentration was also noticed in another salt-tolerant protein DNA Pol-λ from *Arabidopsis*^34^. This structural stability of *Am*_SOD can be linked to the retention of its catalytic activity under highly saline conditions. The ability of *Am*_SOD to exert antioxidant activity under highly saline microenvironment was further confirmed by a functional complementation test where introgression of *Am*_SOD within a *sod* double mutant of *E. coli* conferred tolerance to salt as well as oxidative stress. In addition to salt tolerance, *Am*_SOD also displayed a certain degree of heat resistance. The CD spectra-based melting curve of *Am*_SOD indicates retention of > 85% of its native structural folds at 95 °C. However, in temperature- dependent enzyme assay, *Am*_SOD exhibited a sharp decline in functional activity at 70 °C and onwards. This can be interpreted as even a small fraction of heat-induced conformational change has somehow perturbed the catalytic domain of *Am*_SOD. Thermostable SOD enzymes are predominantly found in peroxisomes. Assuming cytosolic origin, *Am*_SOD is probably an exceptional non-peroxisomal SOD that is resistant to heat denaturation. *Am*_SOD was also found to well tolerate the alkaline pH, which is not very common among the Cu, Zn-SODs. Hence, *Am*_SOD is less resistant to pH-induced conformational changes and metal–ligand leaching. Similar to halophilic *Am*_SOD, some SOD enzymes tolerant to alkaline pH were reported from marine organisms living in a saline environment^35,36^. Altogether, *Am*_SOD can be claimed as a stress-tolerant enzyme with strong free radical scavenging properties. The remarkably high salt tolerance of *Am*_SOD intrigued us to investigate its molecular basis at the residue level. For this purpose, a combinatorial approach consisting of comparative in silico sequence analysis with non-halophilic SODs followed by a mutational study was undertaken. The non-halophilic SODs were selected based on the availability of atomic details of their crystal structures. Many previous reports on extremophilic enzymes claimed that enhanced salt tolerance of a protein is linked to increased accumulation of negatively charged residues (such as aspartate and glutamate) and a decrease in hydrophobic residues on the surface of the protein^28–30^. Such a surface pattern is supposed to facilitate increased hydration even in presence of high salt in the protein microenvironment. Here, we reported the role of 5 residues located in the N-terminal portion of *Am*_SOD that are critical for its halotolerance. These residues are typically located outside of the conserved catalytic domain and were relatively less hydrophobic as compared to their non-halophilic counterparts. This observation was further experimentally corroborated by mutational analysis. It was also noted that not a single residue but the cumulative effect of all the 5 residue substitutions resulted in a drastic fall in salt tolerance. Hence, the salt tolerance of *Am*_SOD can be attributed to a synergistic impact of 5 N-terminal residues that together brought about a decrease in hydrophobic surface area for molecular adaptation. Taken together, the present study presents detailed molecular information on a least characterized stress-tolerant SOD enzyme from an Indian mangrove plant. It also provides a deeper molecular insight at the residue level to understand its mechanism to withstand high salt concentration. It is tempting to speculate that such information will help in crop engineering with better performance in a stressful environment. Such an antioxidant enzyme with noticeably high- stress tolerance will also help to formulate anti-toxicity and anti-aging products of pharmaceutical and cosmetic importance respectively.

## Methods

### Protein extraction

*Avicennia* sp. Leaves were collected from Lothian Wildlife Sanctuary of Sundarban, District South 24 pgs, West Bengal, India. About 100 mg of leaves were homogenized in 2 ml of 200 mM potassium phosphate buffer pH 7.8 containing 0.1 mM EDTA and 1% glycerol for 30 min at 4 °C. The supernatant was filter-sterilized in 0.2 µm membranes (Merck-Millipore).

### SOD assay from leaf extract

1 ml of each reaction mixture was composed of 2.4 µM riboflavin, 0.01 mM methionine, 0.167 mM NBT, 50 mM Na_2_CO_3_, and 200 µl of either leaf extract or buffer (enzyme blank). Samples were illuminated for 15 min and the absorbance was taken at 560 nm against corresponding non-illuminated samples (in dark) set as autozero. SOD activity was calculated as described in^37^.

### Protein purification

50 gm of *Avicennia marina* leaves were homogenized in extraction buffer (0.5 M Tris, pH 7.8; 250 mM NaCl; 50 mM KCl and 0.5% Arginine) at 1:5 (w/v) for 4 hour at 4°C. The clear extract was subjected to 60% ammonium sulfate precipitation. Pellet fraction was reconstituted in 25 mM Bis-Tris buffer pH 5.2 containing 1% glycerol and fractionated in HiTrap-Q^TM^ column (GE Life Sciences) using 0–1 M NaCl gradient. Gel filtration was performed in Superdex S75 column (GE Lifesciences) equilibrated in the same buffer. Protein concentration in each fraction was estimated by Quick Start™ Bradford assay (BIO-RAD). Screening of the fractions was done by riboflavin-NBT based SOD assay and SDS-PAGE.

### Mass spectrometry

The desired band was gel-excised, trypsin-digested as described in^38^ and cleaned up in ZipTip^TM^ (Merck-Millipore). Peptides were subjected to RPLC-MS/MS analysis in a Xevo^®^ G2-XS QT (Waters Corporation) platform. MS/MS spectra were analyzed in the Progenesis QI search engine (Waters Corporation) against the UniProt database using standard search parameters.

### Gene and cDNA cloning

Total RNA and genomic DNA were extracted from 120 mg of *A. marina* leaf using RNeasy and DNeasy Plant Mini Kit (QIAGEN) respectively. Total RNA was treated with RNase-free DNase-I Kit (New England BioLabs) followed by first-strand cDNA synthesis using iSCRIPT^TM^ cDNA kit (Bio-Rad). PCR-amplified full-length gene and mRNA transcript were cloned in pCR^TM^2.1 vector using TA cloning^TM^ kit (ThermoFisher Scientific) followed by transformation into One Shot™ INVαF´ *E*. *coli* (ThermoFisher Scientific). The *Am*_SOD ORF was cloned in pET-22b(+), transformed into DH5α cells of *E. coli* (Bio-Bharati Life Science Pvt. Ltd.). Positive clones were selected on LB agar plate containing 100 µg ml^−1^ of ampicillin followed by Sanger sequencing from Xcelris^TM^ Genomics Labs Ltd., India.

### Purification of recombinant protein

Chemically competent *E. coli* BL21(DE3) Rosetta cells (Bio-Bharati LifeScience Pvt. Ltd., Kolkata, India) was transformed with *Am*_SOD ORF- pET22b+ construct and selected on LB agar plate containing 100 µg ml^−1^ of ampicillin and 34 µg ml^-1^ of chloramphenicol. *Am*_SOD expression was induced using 0.5 mM of IPTG at 16 °C for 12 h and purified under native condition using Ni-NTA agarose beads (QIAGEN) packed in polypropylene-made gravity column following manufacturer’s protocol. Ni-NTA purified protein was refractionated in Superdex S75 column (GE Lifesciences). Fractions containing *Am*_SOD with >95% purity were concentrated in Amicon® Ultra Centrifugal Filter Units, 10 kDa cut-off (Merck-Millipore). *Am*_SOD (1 µg) was run in reducing and non-reducing SDS- PAGE.

### UV–Vis spectrophotometry

The absorbance spectra of 0.8 mg/ml of *Am*_SOD were taken at wavelength from 300 to 800 nm at 25 °C in a double beam Hitachi U-2900 spectrophotometer (Japan).

### Enzyme kinetics

The specific activity and kinetic parameters (V_max_ and K_m_) of recombinant *Am*_SOD were determined by the riboflavin-NBT method as described in^39^. Each 200 µl reaction mixture consisting of 50 mM Tris-Cl pH 7.5, 9.9 mM L-Methionine, 0.57 µM NBT, 1 µg *Am*_SOD, 0.025% Triton-X, and serially increasing concentration of riboflavin (0–0.75 µM) was prepared. Enzyme blanks and non-illuminated sets were prepared for each riboflavin concentration. Absorbance was taken at 560 nm. The specific activity of *Am*_SOD for each riboflavin concentration was calculated by considering 1 unit of SOD enzyme equivalent to a 50% reduction in NBT conversion.

### SOD inhibition assay

The reaction mixtures were prepared as described in ‘Enzyme kinetics’ but with increasing concentrations of either sodium diethyldithiocarbamate trihydrate (0–3 mM) or H_2_O_2_ (0–10 mM) for 30 min. The riboflavin concentration was kept constant at 1.17 µM and specific activity was calculated.

### Zymography

Purified *Am*_SOD protein was run in 10% non-reducing native PAGE. The gel was incubated in 1.26 mM NBT with gentle shaking for 20 min in dark followed by riboflavin buffer (10 mM potassium phosphate pH 8, 126 µl TEMED, and 34 µM riboflavin) with continuous illumination.

### SOD assay under various physicochemical parameters

The reaction mixtures were prepared as described in ‘Enzyme kinetics’ but either with buffers of various pH values or various NaCl concentrations or various temperatures. In each assay condition, the rest of all the physicochemical parameters were kept constant except only the variable one. Comparative enzyme assay with NaCl-treated mutants was performed following the same method for WT *Am*_SOD. The riboflavin concentration was kept constant at 1.17 µM and specific activity was calculated.

### Static light scattering

0.5 mg/ml of *Am*_SOD or the mutant was mixed with various concentrations of NaCl (0–500 mM) and the absorbance at 360 nm was recorded in a UV-Vis spectrophotometer starting from 20 to 180 min at 25 °C.

### Functional complementation test

*Escherichia coli* strain QC774 was transformed with either pET22b-*Am*_SOD construct or empty pET22b+. For control, WT *E. coli* K12 strain with functional *sod* genes was transformed with used. Cells were spread on LB-agar plates supplemented with 100 µg/ml ampicilin. An individual colony from each plate was streaked on LB-agar plate supplemented with 100 µg/ml ampicilin, 0.025 mM methyl viologen dichloride, 0.5 mM IPTG, and either 500 mM NaCl or without salt.

### Circular dichroism spectrometry

CD spectra of 5 µM of either *Am*_SOD or Hel a 6 protein were recorded at 25 °C and 50 nm min^-1^ scan speed in Jasco J-810 spectropolarimeter (Jasco, Tokyo, Japan). The raw CD data was converted in molar ellipticity in CAPITO server^40^. In a step-scan, the CD spectra were recorded by gradually increasing the system's temperature from 20 to 90 °C at an interval of 10 °C. In a descending scan, the system was cooled down to 20 °C and the spectra were recorded once again.

### Fluorescence spectroscopy

0.05 mg/ml of *Am*_SOD in 25 mM Tris-Cl pH 7.8 containing 5% glycerol was separately incubated with 0–700 mM NaCl for 2 h. 2 ml of each sample was taken in a quartz cuvette (4 × 4 mm) and tyrosine autofluorescence was recorded in Hitachi F-7100 spectrofluorimeter (Japan). The excitation wavelength was set at 276 nm and, the emission was scanned from 290 to 400 nm at 30 nm/min speed with 5 nm slit lengths. An average of 3 scans was taken and corrected for control buffer spectra. The maximum emission wavelength was determined by the instrument software with an in-built derivative analysis.

### Fluorescence quenching assay

*Am*_SOD (0.05 mg/ml) was incubated either with or without 500 mM NaCl for 3 h. Excitation was set at 276 nm. Emission of each sample was scanned at 310 nm, first without quencher, and then freshly prepared 5 M of either KI or acrylamide was added in 2 µl increment 10 times. After each addition, the solution was gently pipetted and left for 2 min to attain equilibrium. Quencher concentrations were corrected for ‘dilution effect’. Correction of ‘inner filter effect’ was done using Eq. (1).

1 $$F_{corr} = F \cdot antilog\left( {A_{ex} + A_{em} } \right)/2$$

F and F_corr_ represent the uncorrected and corrected fluorescence respectively. A_ex_ and A_em_ indicate the absorbance at excitation and emission wavelengths, respectively. The quenching data were analyzed according to the modified Stern–Volmer Eq. (2),

2 $${\raise0.7ex\hbox{${F0}$} \!\mathord{\left/ {\vphantom {{F0} {\Delta F}}}\right.\kern-\nulldelimiterspace} \!\lower0.7ex\hbox{${\Delta F}$}} = {\raise0.7ex\hbox{$1$} \!\mathord{\left/ {\vphantom {1 {f\alpha }}}\right.\kern-\nulldelimiterspace} \!\lower0.7ex\hbox{${f\alpha }$}} + {\raise0.7ex\hbox{$1$} \!\mathord{\left/ {\vphantom {1 {f\alpha }}}\right.\kern-\nulldelimiterspace} \!\lower0.7ex\hbox{${f\alpha }$}}K_{sv} \left[ Q \right]$$

where F is the difference between F0 (I_304_ without quencher) and F (I_304_ with quencher); [Q] indicates molar concentration of quencher; fα is accessible fraction of Tyrosine; effective Stern–Volmer quenching constants (K_SV_)_eff_ is equal to fα. K_SV_ values were obtained from the slope and intercept of the linear plot.

### Bis-ANS fluorescence assay

NaCl treated or untreated *Am*_SOD (0.02 mg/ml) was taken in a 3 ml quartz cuvette. A freshly prepared aqueous solution of 300 µM Bis-ANS was added in a 2 µl increment 10 times. After each addition, the solution was gently pipetted and left for 2 min to attain equilibrium. Emission and excitation were set at 490 nm and 390 nm respectively.

### Bioinformatics studies

tBLASTn against NCBInr and nBLAST against the *A*. *marina* genome were performed to identify the transcript and the full-length gene respectively. SOD sequences of *Potentilla atrosanguinea* (UniProt, B2CP37), *Solanum lycopersicum* (UniProt, Q43779), and *Nicotiana tabacum* (UniProt, A0A1S3ZTX1) were retrieved. Multiple sequence alignments were done in ClastalOmega server^41^. Homology modeling of *Am*_SOD was performed in SWISS-MODEL server^42^ using PDB:2Q2L^43^ as template followed by stereochemical quality checking in PROCHECK server^44^. The hydropathy index values of selected amino acids were recorded from^45^. The SASA value of each residue was calculated in GETAREA server^46^.

### Generation of mutants

Mutant constructs in pET22b+ vector were generated by outsourcing from Bio-Bharati LifeScience Pvt. Ltd. (Kolkata, India) as illustrated in supplementary Table S2 and sequenced from Xcelris^TM^ Genomics Labs Ltd., India. The mutant proteins were purified following the same method described for wild-type *Am*_SOD.

### Statistical analysis

Comparison of SOD activity was performed by students t-test in GraphPad prism software V6.1 and significance value was set as *p *< 0.05.

### Ethical statement

All the experimental research done on plants complied with the relevant institutional, national, and international guidelines. Leaf samples from the Lothian Island of Sunderban Biosphere Reserve were collected after obtaining official permission from the Directorate of Forest, Government of West Bengal vide approval letter no. 9(4)/SBR/C-227/17 (Part-II) dated on 09th April 2018. Only a single leaf was collected without destroying or uprooting the plant in presence of forest officials. Plants were identified by corresponding author Kashinath Bhattacharya in consultation with Botanical Survey of India. Voucher specimens were deposited (specimen no. VBH/2019/0012) at the herbarium of department of Botany of Visva- Bharati university.

## Supplementary Information

Supplementary Information.
