# Supplementary material for: Comprehensive characterization and molecular insights into the salt tolerance of a Cu, Zn-superoxide dismutase from an Indian Mangrove, Avicennia marina
Source: Sci Rep. 2022 Feb 2;12:1745. doi: 10.1038/s41598-022-05726-6 (PMC8810880; doi:10.1038/s41598-022-05726-6)
Supplement: Supplementary file 1 — Supplementary Information. [file 41598_2022_5726_MOESM1_ESM.pdf]

Comprehensive characterization and molecular insights into the salt tolerance of a Cu, Zn-superoxide dismutase from an Indian Mangrove, *Avicennia marina*

Rajat Kanti Sarkar[1], Moumita Bhowmik[2], Moumita Biswas Sarkar[2], Gaurab Sircar[1]*, Kashinath Bhattacharya[1]*

[1]Department of Botany, Siksha Bhavana, Visva-Bharati (A Central University), Santiniketan 731235, West Bengal, India

[2]Division of Plant Biology, Bose Institute, Kolkata 700009, West Bengal, India

*correspondence: gaurab.sircar@visva-bharati.ac.in, kashinathb23@rediffmail.com

Supplementary figures S1 to S2

Supplementary table S1 and S2

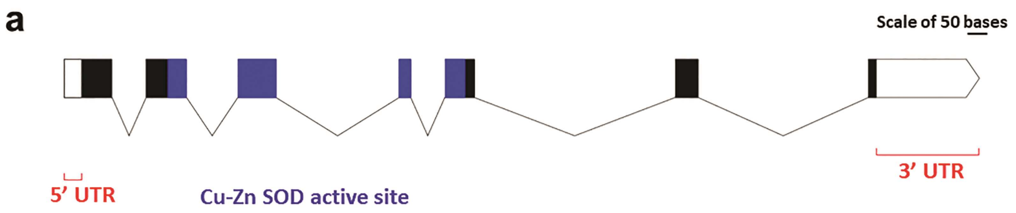

b

5'GTCGCTGCTTTCTAACGCGCAACCGGGGTGTCCTGAGATCACAATCATGCCGAAGGCTGTCGCCGTACTTAGCAGTAATGAGGGCGTTAGGGGCACTGTTTACTTCACACAAG
AAGGAGATGGtacttttcatttccattaaatcaagtgttctgcctatatttattaaattcggacagatgataaccctcgcttctttcatgcttcaggGCCCACTACCGTAACTGG
AAACCTTTCTGGCTTTAAATCTGGTCCCCATGGCTTTCATGTGCATGCCCTTGGTGACACCACCAATGGCTGTATGTCAACTGGTaatttctcctgcacaaatttttattggctc
catcaagaaatatctgttggctgcattggcatggttttctatttaacctttgccaattgatttcacgacatttcatattcttttaatttcaatatgtaggtCCTCACTTCAATCC
GGCTGGCAAAGACCATGGTGCTCCAGAAGATGAGGTTCGCCATGCTGGTGACCTCGGTAACATCACTGTTGGAGAAGATGGTAattacctttaaaattttgtgcattcttgtatt
ttgaattgtatgttaggatatatttctttgtcttgtgtaactggaactgaaagtcagagctctagttcccaattaaaatgaaataaagaagaaactgaagatcacatgcttttgg
cttctatgtttttttgaagttctgtttattgggcttcatgtctttttctgtgctgaaagttcatcttgatactgactgctaccccacttgtattatgctacattgtggtcccataa
tccaattgtaattaatatatctccttttttactgctttctcaatgtttcaggtaCTGCTGCCGTCAACATTGTTGACAAGCAGgtctatttcttcactaccatcttctgtaaaaat
cttaagttgtttctggctttaaagtgtcataagcatttttacattgttttgcagATACCACTTTCAGGACCACATTCCATAGTTGGAAGAGCTGTAGTTGTCCATGCCGATCCTG
ATGATCTTGGAAAGggtgtgtgtcccatatttcctttgctgattatgctaacctctgtacttatcaatcccattaatctaaactagttatcttactgctagaaattattaagcat
aatgtgaaattaatttaattgcttgaggggaagtatgggagaggacatttagatattatggtcaccagttggattgacaaacaagttactggcaacctttagtgtgcttttgct
attgtttgtagcaactgctaagtggatctctgtctgcaagaacttgatgtcaaaaatttttttatttaataaagtgggattatatgaggctgacttcatgagacatcagtaatatt
atgtgtgtttctcatttgtttggtttaaaattatttttgattggctgggccatggacaaagtaaggtataaaatgctgcaacatttgagtgatatgcttttaggaattgtatttg
ttttgaaatcaagtgctttgtgtttcgaaatatatgcaatcatctcatgactgagtttgttgccctaGGCGGACATGAACTAAGCAAAACTACTGGAAATGCTGGTGGAAGAGTT
GCTTGTGGTAaggaaaactgtcatagatctaataatgtcattcacatgccttgatgcatgagagatgttctggagtgccaagttttaaatttattcatgctgcttcacattgata
ggcataattaagatatatttgcttgttttaaggctttctgtaaggattccgctaatcttgtagaaacaagtattctgtcattgcaaagaactaagaaaattagttctttatagct
ttcatgaagagtgcctaaaacccatgttggtacgaaagatagttttgattcaaatttcaggcacttgtgaagaaatgatgattcacttgcttatcgaattatatagcgacttttg
ccacaacgatgtcgtggcttataattgagtgcaaacgaatttgaaaaaagcattaggcagctgcagtctccttacactttttgttaatgttatgcaggtaTCATTGGTCTTCAGG
GCTGAACAACCCTGGCTGGGCACTCTTGACTTTTGTACTCTTTCATCATTACCCAGTCGCTTGTAAGCANAGTCTCGGATGAATGTGATTTGCTTGCTATCAATAAAGGCTAGTT
GGAATGGATGGGTCATGTGTGTAATAGTACCCTTGATGCCTAATGAGAGTTTGATATTATATGCTATTCTGTGACTGAAACCCATATGTGATCAATGCGTTCCCACATCATCTTT
GCTCCTAAAAAAAAAAAAAAAAAAAAAAAAAAAAAAAA3'

Supplementary figure S1: ***Am*_SOD gene**. (a) Diagrammatic representation of the domain architecture of full-length *Am*_SOD gene. White, black, and blue blocks represent untranslated regions (UTR), exons, and catalytic domain respectively. Lines represent introns. (b) Nucleotide sequence of full-length *Am*_SOD gene. Shown UTR, exons, introns, and catalytic domain in red, black uppercase, black lowercase, and blue uppercase respectively.

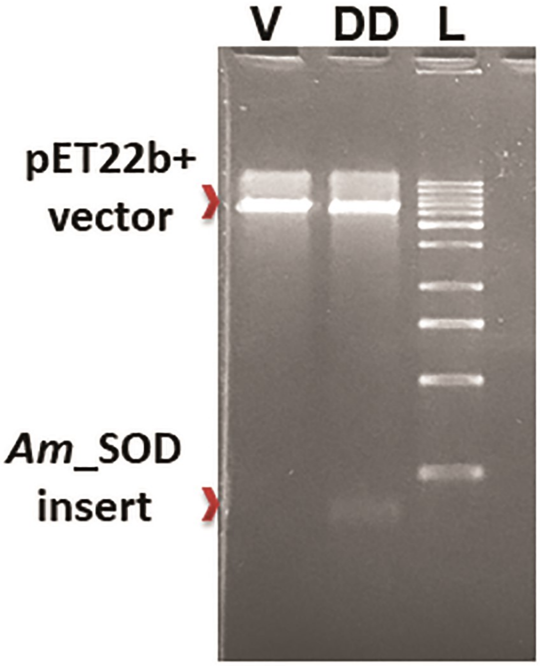

Supplementary figure S2: **Cloning of *Am*_SOD cDNA.** 1.2% agarose gel showing cloned cDNA coding for the full-length *Am*_SOD as 459 bp insert released from the pET22b+ vector after double digestion with NdeI and XhoI (lane DD). Linearized pET22b+ vector was run as control (lane V). Right margin showing 1 kB DNA ladder (lane L).

Supplementary table S1: Results of LC-MS/MS analysis of trypsin-digested peptides for the

Peptide	Theoretical m/z	Observed m/z	Score	Sequence coverage (%)	UniProt ID	Significance
AVAVLSSNEGVR	1201.345	1201.688	5.385	7.895	Q9AXH2	P< 0.005
GTVYFTQEGDGPTTVTGNLSGFK	2376.562	2377.001	4.998	15.132		
DHGAPEDEVR	1124.132	1124.802	5.627	6.579		
GGHELSK	726.787	726.058	6.237	4.606		
TTGNAGGR	732.751	733.002	5.229	5.264		
QIPLSGPHSIVGR	1360.579	1360.821	6.008	8.553		

identification of *Am*_SOD.

.

Supplementary table S2: Generation of *Am*_SOD mutants by point mutation on critical residues responsible for salt-tolerance.

Point Mutants	Critical residue substitution	Hydropathy index value	SASA value (A+B chain)	Mutagenesis method
Single	Pro^{2} – **Val**	-1.6	79.18	Site directed mutagenesis by Mutagenic primers using *Am*_SOD construct as template
Single	Asn^{11} – **Ser**	-3.5	311.574	
Single	Arg^{15} – **Ala**	-4.5	266.909	
Single	Tyr^{19} – **Leu**	-1.3	147.538	
Single	Pro^{41} – **Leu**	-1.6	121.612	
Multiple	Pro^{2}Asn^{11}Arg^{15}Tyr^{19}Pro^{41} – **ValSerAlaLeuLeu**	NA	NA	Synthetic g-block codon optimized for *E. coli* from IDT Inc. (USA)

Footnote for table S2: The critical residues of Am_SOD are shown with corresponding position in *Am*_SOD sequence as superscript. The substituted residues from non-halophilic SOD are shown in bold. Hydropathy index and Solvent accessible surface area (SASA) values of the critical residues of only *Am*_SOD are shown (Those of non-halophilic SOD are not shown). Combined SASA values from both chains (A and B) of the dimer are shown.
